# The Role of Corporate Social Responsibility and Corporate Image in Times of Crisis: The Mediating Role of Customer Trust

**DOI:** 10.3390/ijerph18168275

**Published:** 2021-08-04

**Authors:** Chih-Cheng Chen, Asif Khan, Tanaporn Hongsuchon, Athapol Ruangkanjanases, Yen-Tzu Chen, Ornlatcha Sivarak, Shih-Chih Chen

**Affiliations:** 1 Department of Marketing and Distribution Management, College of Management, National Kaohsiung University of Science and Technology, Kaohsiung 824005, Taiwan; volvic@nkust.edu.tw; 2 Chulalongkorn Business School, Chulalongkorn University, Bangkok 10330, Thailand; athapol@cbs.chula.ac.th; 3 Department of Information and Learning Technology, National University of Tainan, Tainan 70005, Taiwan; yentzu@gm2.nutn.edu.tw; 4 Mahidol University International College, Mahidol University, Nakhon Pathom 73170, Thailand; ornlatcha.siv@mahidol.ac.th; 5 Department of Information Management, National Kaohsiung University of Science and Technology, Kaohsiung 824005, Taiwan; scchen@nkust.edu.tw

**Keywords:** corporate social responsibility, multi-dimensional CSR, corporate image, customer trust, emerging economy, times of crisis, smart PLS, mediation

## Abstract

The purpose of this research is to empirically examine relationships between a multi-dimensional set of corporate social responsibility (CSR) initiatives, numerous dimensions of customer trust, and corporate image in an emerging economy. It also analyzes the mediating effect of customer trust on the relationship between CSR and corporate image. This study focuses on two of the most well-known hotel chains situated in Pakistan. Close-ended, self-administered questionnaires were circulated amongst a total of 300 hotel customers. The research data was analyzed using a partial least square-structural equation modeling (PLS-SEM) model. The results revealed that economic, legal, and ethical CSR significantly impacted corporate image, while philanthropic CSR did not affect the corporate image. However, economic, legal, and philanthropic CSRs were found to be in a significant relationship with customer trust, while ethical CSR was not in a significant relationship with customer trust. Finally, customer trust fully mediated the relationship between economic and legal CSR with corporate image, whereas it partially mediated the relationship between ethical and philanthropic CSR. This study is unique from earlier CSR research based on an assessment of the connection between CSR dimensions and corporate image to examine customers’ trust in an emerging economy, especially in times of crisis.

## 1. Introduction

Corporate Social Responsibility (CSR) describes how a company manages its industry and takes responsibility for its social impact. Corporate social responsibility encompasses various characteristics like economic dependence, legal conformity, ethical requirement, and societal influences [1]. CSR has been suggested to benefit the reciprocally advantageous long-term and reliable relations with its participants [2]. Customers are key participants of a corporation, hence, exploring corporate social responsibility from the perception of customers has progressively drawn the attention of investigators and experts, especially in the service industry [3]. Furthermore, times of crisis are described as the complex challenges faced by the companies. These challenges are mostly related to the strategic focus of the companies in times of economic downturns. According to public opinions, financial challenges and profit-making ability are deemed as the core aspects of a company in times of crisis. In contrast, other social challenges and responsibilities of the business are important but ignored in previous research, however, these challenges are supported by some of the previous research papers [4,5,6].

Consequently, corporations are expected to fulfill ethical and social responsibilities rather than utilizing them as a differentiating approach to achieve organizational authenticity [7]. The growing awareness and insight of CSR initiatives have driven CSR activities as a competitive strategy between firms. 

Corporations consider the use of CSR strategies crucial in determining organizational objectives for enhancing corporate image (CI) and profitability by recognizing the need and significance of CSR activities [8]. Customers are extra positive to corporations that enthusiastically publicize their corporate social responsibility programs as compared to those corporations that would not promote their CSR activities [9]. Corporations’ CSR methods have significantly influenced corporate image in a positive manner and caused the largest growth in market share [10]. Therefore, many corporations have utilized CSR activities as a distinctive management approach. A corporation’s CSR execution can, as a result, have both a negative and a positive impact. As CSR becomes essential, both as a key academic course and a component of the corporate plan, customers are following up on corporations’ commitment in CSR more enthusiastically than earlier. The corporate image that is achieved as a consequence from CSR procedures may possibly enhance customer social accountability conduct and the association among the company and customers [11]. 

Customers will participate actively like the firm’s employee if he/she has an optimistic image of the corporation [12]. Corporate social responsibility additionally operates as a marketing instrument since it provides to set a constructive corporate image [1,13,14]. Numerous investigations have recognized the growing association concerning corporate image and corporate social responsibility initiatives. Corporate social responsibility is a crucial component in enhancing corporate image. Corresponding to a study performed in Korea confirmed that corporate achievements of financial and legal corporate social responsibility policies had an immediate and significant impact on the corporate image [15]. Moreover, a significant association between corporate social responsibility and the corporate image was exhibited to impact market share [16,17]. To be precise, companies’ legal and ethical status concludes in a constructive corporate image of the company. Furthermore, companies are expected to engage in socially responsible endeavors by their customers, and the customers express their gratitude to the companies’ behaviors by purchasing their products [18]. A few research scholars claimed that customers who believe admirably of companies are highly prone to engage in long-term relations with those companies [19].

Corporate social responsibility has a significant influence on business interactions with customers, and disreputable marketing conduct negatively affects the attitudes of customers, social conduct, and satisfaction. The business and its customers can be deemed corporate allies and their relationships are significantly influenced by their belief in mutual trust [20,21]. Furthermore, services industries have to handle several additional interactions with their customers in comparison to fast-moving consumer goods (FMCG) industries [21]. Therefore, in a service commerce’s competitive framework, businesses can not merely introduce trust via truthful, and honest communication initiatives, but they also need to produce trust at each particular interaction phase, which forms a positive customer experience in the minds of customers, specifically when customers communicate with the frontline staff [22]. Hence, service industries have to build and represent their dedication to corporate social responsibility policies and programs thoroughly at all the various interaction points creating the customer experience [23]. In addition, the innovative digitalized and linked atmosphere additionally presents customers the likelihood of networking considerably more immediately with businesses [24,25]. Moreover, corporate social responsibility’s relationship with customers and company’s characteristics [26] causes mediation in the relationship between corporate social responsibility programs and company’s assessments [27]. Customers’ trust related to a corporation and its products is strengthened by the effective execution of CSR initiatives. [27]. Although customers’ trust is an important factor to be examined, yet limited research has been conducted to explore the role of customers’ trust within the framework of CSR. This research has utilized the rising multi-dimensional view of this construct. 

To address the previous research inadequacies of CSR related to the measurement and conceptualization of CSR and customers’ trust, this research study provides and empirically examines the research model that integrates the relationships between a multi-dimensional set of CSR initiatives namely legal, ethical, economic, and philanthropical CSR proposed by Carroll [28] and a numerous dimensions of customer trust containing integrity, experience, and benevolence, based on trust theory [29] and corporate image in an emerging economy. This research attempts to address at least four key research gaps related to CSR. First, we examine how CSR helps support corporate image. Second, we explore the relationship of CSR with customer trust. The third research gap covers the impact of customer trust on corporate image. This contributes to the fourth research gap, which is built on prior work by assessing the mediating role of trust on CSR to illustrate the influence of corporate social responsibility on corporate image. 

## 2. Theoretical Background and Hypotheses Development

### 2.1. Corporate Social Responsibility (CSR) and Corporate Image (CI) 

Carroll [28] conceptualized the CSR model by proposing to integrate four accountabilities: economic (delivering required products and services), ethical (implementation of the ethical conduct), legal (compliance to policies), and philanthropic (participating in volunteer activities). Furthermore, Carroll [28] stressed that the advantages gained by the company from CSR might also impact its stakeholders. A stakeholder can be described as “an entity or a group which might impact or is impacted by the fulfillment of the corporation’s objectives” [30]. 

CI, achieved by the company’s performance towards CSR initiatives, can be defined as the subjective view of customers related to the performance in terms of the stakeholders’ societal concerns [8]. Corporate image is characterized as the reaction to the amount of trust, concepts, and thoughts the communities have in the direction of a corporation [31]. Furthermore, it is also related to the stakeholders’ perception regarding the necessary measures taken by the company for the betterment of its stakeholders. Corporate image is an intangible asset that can improve customers’ behavior intention including customers’ need fulfillment, commitment, and repurchase intention to endorse [8]. Corporate Image is significant for achieving trade gains, hence the significance of social accountability in terms of establishing an optimistic image of a corporation is enhanced [32]. CSR integrates CI in the minds of customers and a positive corporate reputation is achieved. A corporation’s reputation is a critical tactical source for achieving viable gain [33]. On the Contrary, not implementing corporate social responsibilities can eventually cause an adverse impact on the company. Previous research suggested that companies that honestly take measures for their social responsibilities and provided an optimistic image to their stakeholders were further expected to generate strong financial outcomes [34]. Effective CSR implementation can be achieved by the active simultaneous participation of external and internal stakeholders; hence it can enable a company to forecast and take benefit of rapidly changing social environments and beliefs. Service firms, like any other company, are required to be in harmony with sociocultural, socioeconomic, and environmental activities. Earlier studies confirm the relationships among corporate social responsibility and corporate image by verifying that a company’s corporate social responsibility methods significantly impact the company’s image, consumer attitudes and the company’s ([8,11,35]. These initiatives additionally enhance relations among the customers and the company [10,36]. Park et al. (2014) have claimed that a company’s realization of legal and economic corporate social responsibility activities had a significant impact on a company’s reputation, however, both philanthropic and ethical corporate social responsibility activities had a significant impact. Plewa et al. [11] presented that acquaintance of customers with a company’s Corporate Volunteering (CV) initiative is significantly associated with customer insights of the corporate image and customer acknowledgment of other placed motivations, furthermore, customers’ perceptions of a company’s corporate social responsibility reputation are significantly associated to the company’s image. Consequently, it can be inferred that corporate social responsibility initiatives can improve a firm’s corporate image for shareholders. In the research studies related to hospitality, Kim et al. [10] uncovered that each element of corporate social responsibility (ethical, economic, philanthropic, and legal) with the exception of legal accountability had a significant impact on the CI in the casino business. Ghaderi et al. [37] confirmed that each aspect of corporate social responsibility has direct and positive effect on the performance of the hotel. 

Previous research on corporate social responsibility uncovered that corporate social responsibility has a significant association with corporate image, suggesting the utilization of corporate social responsibility as the instrument to improve corporate image. In additional words, when service suppliers operate well at corporate social responsibility, customers will create a constructive impression of them. This mutual association has been endorsed by current literature [38,39,40,41]. Popoli [42] also assert that corporate social responsibility has a significant impact on improving customers’ views on a corporation’s image. According to Hillenbrand, Money, and Pavelin [43], an improvement in the company’s image might be a consequence of a company’s philanthropic contributions, in that case, if the initiative and motivations related to the donation are perceived as optimistic. Additionally, for a company to achieve a responsible corporation reputation, the company is necessary to be viewed as a well-behaved corporation by its stakeholders (i.e., being professional in conducting company’s interests, transparent, sustainable, and reduce adverse social effects) and with decent intentions (i.e., being reliable, honorable, and honestly care about the people). 

B. Kim, Lee, & Kang [44] hypothesized that corporate social responsibility performances have a significant impact on company reputation in the framework of tourism. Lee, Kim, & Ham [45] uncovered that CSR affects brand trust and brand image. Thus, it is evident from the empirical and theoretical perspective that if the CSR initiatives are perceived optimistically by the stakeholders, they might be more favorable in leading towards the corporate image. Hence, established on Carroll’s [28] four corporate social responsibility aspects and the previous study, we recommend the following hypotheses.

**Hypotheses** **1a** **(H1a).** *Economic (CSR) positively influences corporate image (CI) in times of crisis.*

**Hypotheses** **1b** **(H1b).** *Legal (CSR) positively influences corporate image (CI) in times of crisis*.

**Hypotheses** **1c** **(H1c).** *Ethical (CSR) positively influences corporate image (CI) in times of crisis*.

**Hypotheses** **1d** **(H1d).** *Philanthropical (CSR) positively influences corporate image (CI) in times of crisis*.

### 2.2. Corporate Social Responsibility and Customer Trust

Customer trust is the topic of significant efforts to describe it. For Barber (1983) trust is defined as a group of “socially realized and verified beliefs” that individuals have of other individuals or organizational bodies. Trust builds as a consequence of a company’s belief that the trustee is trustworthy, sincere, and compassionate [46]. According to the customer’s perspective trust can be defined as the customer’s belief that a company will operate based on the expectations concerning its capability, goodwill, and integrity. Trust is perceived as a multidimensional concept [47], though most of the time it has been theorized and operationalized as a unidimensional global concept [27,48]. The customers and brand can be deemed corporate partners, whose relationships are impacted by their individual opinions of shared trust [20]. Consequently, trust can be described as the belief that every corporate partner should perform with reliability and trustworthiness throughout their collaborations. Conventionally, numerous researchers have recommended reliability and integrity as essential dimensions of trust. Moreover, corporate partners are prone to build trust by staying trustworthy, altruistic, and benevolent [21]. In this research study, the three-dimensional description of customer trust proposed by Mayer & Davis [47] is adopted, which comprises integrity, expertise, and social benevolence. 

The viewpoint regarding the dearth of devious conduct between corporate partners is likewise vital for trust advancement, as is the viewpoint that the brand works reasonably, accountably, and conscientiously for its customers [20]. Various researchers have demonstrated that customer assessments that the business performs in a socially accountable way are significantly associated with customer trust in that business [49,50,51]. Analyzing customer experiences of corporate social responsibility, Vlachos et al. [52] recommended a framework linking customer opinions of the business’s motivations for accepting corporate social responsibility measures to customer trust. Generally, socially accountable conduct is crucial, since a business, which is recognized as socially conscientious is further expected to be trustworthy by its customers [51]. Similarly, in the service industry, García de los Salmones et al. [53] discovered that the ethical conduct of a customer services business has a significant influence on customer trust. Furthermore, Choi and La [54] uncovered that corporate social responsibility has a significant effect on customer trust in several service industries including financial services, restaurants, and airlines. In the hotel business, Martínez and Rodríguez del Bosque [50] offered statistical proof of a significant impact of corporate social responsibility on customer trust. 

A community’s ethical view regarding the company is vital to construct a reliable connection, hence companies are more motivated to engage in socially responsible programs to express their dedication to the community [55]. According to Kim et. al [56], CSR is found to be one of the top approaches to stimulate trust in customers. Trust can be described as “the belief of ethically acceptable behavior” [57]. Firms, engaged in CSR initiatives, discovered CSR to deliver advantages like customer loyalty, customer satisfaction, good image, and high market value [48]. Performing company functions in an ethical fashion affects the complete image of the company’s products or services and enhances customer trust [58]. An optimistic view of the company concerning CSR will impact consumer trust. Trust in a service industry is greatly linked to experiences of service provider’s honesty, integrity, and morality [54]. Paine [59] asserts that devotion to moral norms offers the foundation for trust that improves to create repute, and strengthens the supply of value services. Similarly, ethical conduct by the staff of a business has a significant influence on customer trust. 

**Figure 1:**
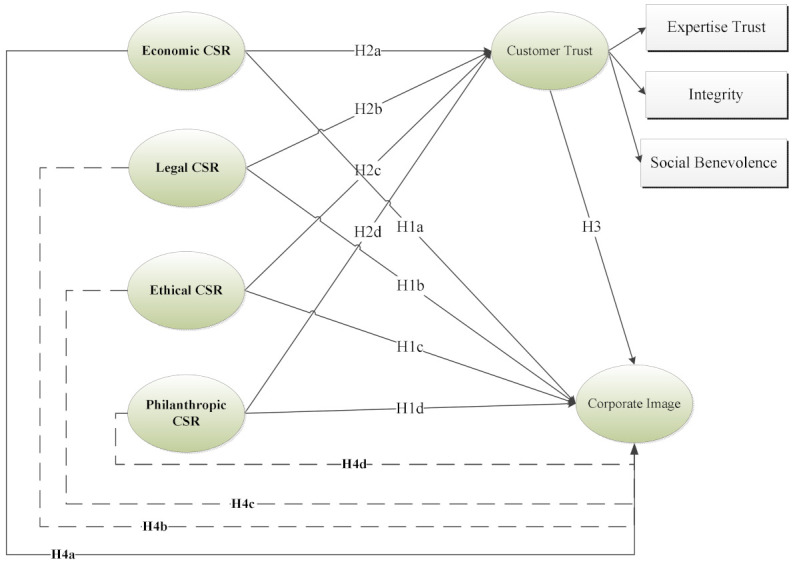
Theoretical Framework.

These research studies provide evidence that CSR initiatives must place trust in the minds of customers’ regarding the company’s societal concern intent to engage in CSR initiatives to establish a positive impact on corporate image. In the model (Figure 1), the three types of trust are positioned as mediators of the effects that the four types of CSR initiatives have on corporate image.

**Hypotheses** **2a** **(H2a).** *Economic (CSR) positively influences customer trust in times of crisis.*

**Hypotheses** **2b** **(H2b).** *Legal (CSR) positively influences customer trust in times of crisis.*

**Hypotheses** **2c** **(H2c).** *Ethical (CSR) positively influences customer trust in times of crisis*.

**Hypotheses** **2d** **(H2d).** *Philanthropical (CSR) positively influences customer trust in times of crisis*.

### 2.3. Customer Trust and Corporate Image

The collaboration among employees and customers to produce an image must turn out to be an attractive study. According to a research finding, service companies like hotels were indicated as brands. Consequently, the values of a company are characterized by brands. Corporate image is an element of the argument regarding brands [60]. Based on this argument it can be underlined that whenever an employee presents a brand via collaborative initiatives with customers, the view regarding the company’s image can be influenced. Service companies like hotels perform corporate image by agents of supervisors in conveying and delivering services to customers [61]. The managers and employees of the company deliver values to customers which in turn becomes optimistic impressions and beliefs [61,62].

Trust is an essential concept in several academic disciplines, particularly in theories related to marketing and organizational management. In a provided exchange association, trust is described as the degree of trustworthiness ensured by the service provider to the customer, and it was demonstrated to be a vital aspect in retaining a constructive connection between the business and the customer in marketing. Customer trust is regarded as customers’ trustworthiness about a business that is defined by a business’s ability to genuinely fulfill the promises made to the customers. Since the corporate image is created by trustworthy initiatives of a company, hence, trust is deemed as a critical element in the success of a business. Providing agreed value is crucial to developing an optimistic repute. As trustworthiness is fragile, there is a great effort to recapture trustworthiness after it is lost [63]. Trust was additionally discovered to be the antecedent component of corporate image [15,63]. 

The hotel industry’s psychological image can be described by several methods, comprising trust, favorability, status, and recognition. Corresponding to the opinion of the hotel managers the corporate image is exhibited by the customers’ trust in the hotel [60]. It is also found that the hotel management finds trust to be a major factor for attracting visitors to the hotel. Hence, it implies that each hotel should be able to develop a psychological understanding of the attitudes of customers so that they can be motivated to experience the same brand one more time [64,65]. Privacy and safety are found to be vital factors in determining trust in a hotel. Hence, managers at a hotel are found to be paying close attention to ensuring the privacy and security of customers [60]. 

Based on the following discussions it can be proposed that:

**Hypothesis** **3** **(H3).** *Customer trust has a significant association with the corporate image in times of crisis.*

### 2.4. Mediating Role of Customer Trust

If a company is trusted by the customers, the customers would be more committed to facing risks, and therefore, this risk-taking conduct would have certain constructive consequences on the business [29]. A company’s CSR initiatives are found to be an important factor in deciding the perception of customers’ trust in the company. By participating in CSR programs, a business demonstrates to customers that it is concerned regarding the outcomes of its actions (benevolence) and supports some key values, such as reducing its adverse impacts on the community and the ecosystem (integrity) [66]. 

Consumer trust is influenced by the presence of principles that the customers and the company communicate. Concerning CSR programs, this conduct offers knowledge about business personality and principles, and it is valuable for boosting overall trust in the direction of the company [50]. As Hosmer [67] reveals, by introducing ethical and trustworthy values into businesses’ tactical decision-making practices companies can improve the trust of all shareholders, as well as customers. The view that a business is moral and trustworthy promotes trust-based relations established in the principle that each exchange partners’ activities will be plausible ahead of any legal or contractual limitations [51]. In an endorsement of this viewpoint, Pivato et al. [48] additionally recommended that “the establishment of the trust is one of the greatest direct outcomes of a business’s societal functioning” or the direct or highly immediate consequence of CSR actions. Social relationships have been implemented primarily via customer trust in marketing relationship studies. Trust has been identified as the presence of a connection where one associate that is the customer in this study, trusts in the other associate that is the hotel in this study in terms of integrity and reliability [3,68]. Additionally, customer experiences of trust correlate to the business’s thoughtfulness and reliability beyond its competence [69]. Therefore, it has been indicated that a business’s real commitment to corporate social responsibility actions concerning its customers is useful for enhancing overall trust and decreasing distrust for the business [52]. Moreover, studies by Nicki [3], Swaen and Chumpitaz [51], and Garcı´a de los Salmones et al. [53] discovered a significant correlation between a business’s corporate social responsibility and customer trust in the service industry indicating that trust is the direct or most immediate result of a business’s CSR initiatives. 

According to the results of the study by Triatmanto et al [60] respondents approved that the psychological insight of the corporate image was certainly perceived as trust, awareness reputation, and favorability. Trust was found to be the most vital indicator from the four mentioned indicators, which was viewed optimistically by the respondents, indicating that in the hotel business, customers are more involved with hotels that are perceived to be trustworthy. Safety and privacy offered by a hotel can earn the trust of a hotel [70,71]. According to the model (Figure 1), the customers’ trust is placed as a mediator of the impacts that the CSR programs have on corporate image:

**Hypotheses** **4a** **(H4a).** *Customer trust mediates the association between economic CSR and corporate Image*.

**Hypotheses** **4b** **(H4b).** *Customer trust mediates the association between legal CSR and corporate Image*.

**Hypotheses** **4c** **(H4c).** *Customer trust mediates the association between ethical CSR and corporate Image*.

**Hypotheses** **4d** **(H4d).** *Customer trust mediates the association between philanthropic CSR and corporate Image*.

## 3. Methodology

### Sample and Procedure

This study focuses on two of the most well-known hotels situated in Pakistan. A total of 300 hotel customers staying in Peshawar and Islamabad cities were selected. Close-ended self-administered questionnaires were circulated amongst the customers. Instead of requesting respondents merely whether they approve an opinion statement, the research paper used Likert scale items, in which the customers were requested how strongly they approve or disapprove with it, typically on a 7-point measure from 1 (=strongly disagree) to 7 (=strongly agree), with 4 demonstrating a neutral category.

A pretest will be conducted by a sample of 50 customers located in Peshawar city. Corporate social responsibility will be assessed by items recommended by [10] however, the items to measure Corporate image was applied from Chowdhury et al [72] and Kim et al [10] research study. Furthermore, McKnight et al [46] methodology was used to measure customer trust. The questionnaire is shared in the Appendix A. The hypothesis of this study will be tested using a structural equation model.

## 4. Data Analysis

Partial least squares (PLS) measurement was conducted using two measures. In the preliminary phase, the reliability and validity evaluation was conducted, whereas, in the next phase, the path coefficients and the structural model’s explanatory power were analyzed and evaluated. The purpose of the two phases mentioned was to verify the construct’s reliability and validity, including checking the relation among the constructs [73,74]. PLS has been applied and deemed as the finest tool for demonstrating the causal collaboration among constructs and consequently can handle model measurement items and construct variables simultaneously [75]. Moreover, because PLS uses simpler factors to measure the randomness and normality of the variables, it is found to be perfect for reviewing the association among constructs in the distribution of the irregular results. It also shares the advantages of assessing prediction models possessing dynamic qualities [76]. PLS was therefore considered to be more appropriate for this study as compared to other SEM analysis methodologies to assess the relations between construct variables, eradicate measurement errors, and prevent collinearity.

### 4.1. Convergent and Discriminant Validity

The associated external model measurements contained the internal consistency of every measurement item, the convergent validity, reliability, and differentiating validity of each layout. Employing an applicable loading of queries, the reliability of the results was analyzed. Factor loading’s threshold value of individual reliability was considered to be 0.6 [77]. Individual reliabilities were based on this threshold value. All the observed variables in the study adhered to the criteria and went through the elimination process. Table 1 shows the composite reliability of each construct. The composite reliability (CR) values of all the variables were found to be higher than 0.7 [78] and suggested that the construct was adequate.

The factor loading, composite reliability, and the average variance extracted AVE factors for all construct variables were studied. A construct would be considered to possess an acceptable convergent validity if its related indicators were possessing values greater than 0.5 [79]. Table 1 reveals that the AVEs in this analysis is between 0.591 and 0.835 for hypothetical construct variables, suggesting that there is substantial convergence.

Discriminatory validity was utilized as a measurement to analyze the discrimination among different constructs and items. According to the results demonstrated in Table 2, the factor loading of each item exceeds the factor loading of other constructs, hence indicating possessing a fair discriminant validity [80].

The quality assessment of the model was done by measuring the Goodness of Fit (*GOF*), which was analyzed using Tenenhaus et al. [81] proposed model, calculated as follows:

$$GOF=\sqrt{\bar{AVE}}\times \sqrt{\bar{R^{2}}}=\;\sqrt{0.726\times 0.697}=0.711$$

Corresponding to the above-mentioned formula *AVE* is measuring the average variance, while *R* square is the coefficient of determination. According to the outcome, the *GOF* is 0.711, which achieves the 0.28 cut-off standards for a large impact size [82].

Furthermore, standardized root mean square residual (SRMR) is characterized as the distinction between the studied relationship and the framework indicated relationship matrix. Hence, it lets evaluating the mean of the differences among studied and anticipated relationships as an indisputable scale of model fit standard. Consequently, if the model has an SRMR value of less than 0.08 it has a good model fit [83]. Henseler et al. [84] propose the standardized root mean square residual as a GOF gauge for PLS-SEM, which can be utilized to prevent model error. Additionally, Bentler-Bonett Normed Fit Index (NFI) is a phased gauge of GOF for a research model that is not influenced by the model’s number of factors. The Normed Fit Index is the difference of 1 and Chi^2^ divided by the model’s Chi^2^. Thus, the Normed Fit Index scores values between the range of 0 and 1. Normed Fit Index values exceeding 0.9 normally signify a satisfactory fit. According to the findings of smart PLS, the value of SRMR and NFI were 0.034 and 0.951, respectively. Given that SRMR < 0.08 and NFI > 0.90 the model is significant.

### 4.2. Empirical Results

The hypothesis of this study was analyzed by using the internal PLS model. The path coefficients are the direction and power of the correlation among the constructs that signify cause and effect among the assessed latent and variables. Furthermore, the model’s analytical capability can be demonstrated by the value of the R square. The degree of all the path coefficients was analyzed by using the bootstrapping approach. Re-sampling the data was used to improve the estimated limit value [85]. Consequently, this approach was adopted by the research study to examine the significance among variables. The findings of the study were divided into two models; model A of the study analyzed the relationship between CSR and corporate image without the mediating effect of customer trust, while model B integrated customer trust as a mediating variable. 

**Figure 2:**
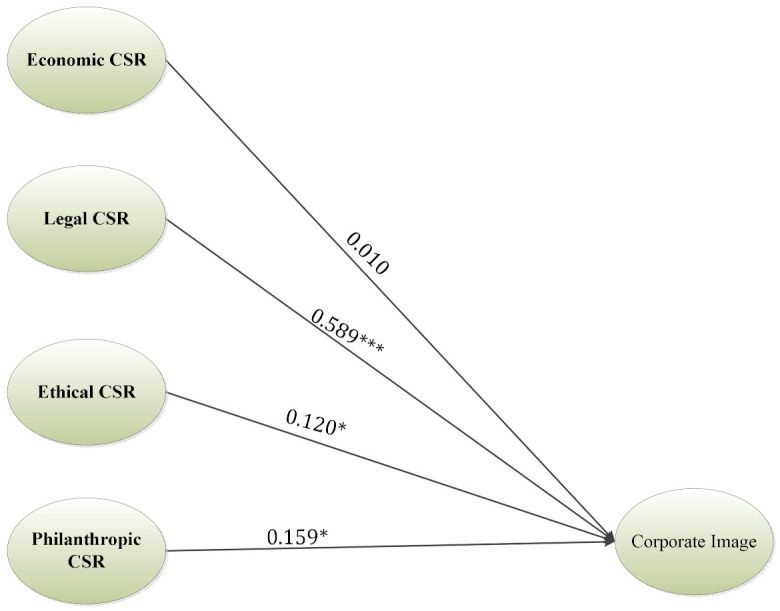
Results of the inner model A. Note: *** *p*-value < 0.001, * *p*-value < 0.05.

According to the findings of model (A) highlighted in Table 3, and Figure 2, legal, ethical, and philanthropical responsibilities of CSR were all found to have a positive influence on the corporate image supporting H1b (β= 0.589, t-value= 7.805), H1c (β= 0.120, t-value= 2.191), and H1d (β= 0.159, t-value= 2.304), while not supporting H1a (β= 0.010, t-value=0.297), indicating that economic responsibility has no significant relationship with corporate image.

**Figure 3:**
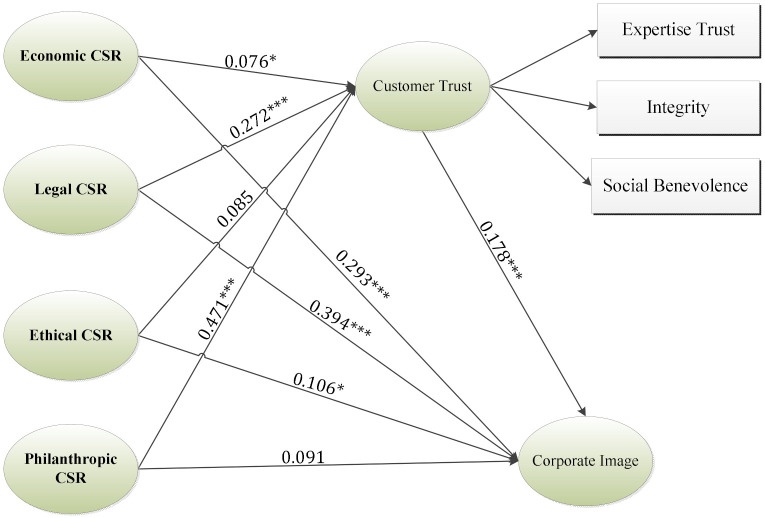
Results of the inner model B. Note: *** *p*-value < 0.001, * *p*-value < 0.05.

According to the findings of Model B highlighted in Table 4, and Figure 3, economic, legal, and philanthropical responsibilities of CSR were all found to have a positive influence on the corporate image supporting H1a (β= 0.293, t-value=6.80), H1b (β= 0.394, t-value=6.198), and H1c (β= 0.106, t-value=2.332), while not supporting H1d (β= 0.091, t-value=1.323), indicating that philanthropic responsibility has no significant relationship with corporate image. According to the statistical results, the relationship between CSR and corporate image has an incremental significance by including customer trust as a mediator.

The economic, legal, and philanthropic responsibilities of CSR influence customer trust, therefore H2a (β = 0.076, t-value = 1.936), H2b (β = 0.272, t-value = 3.627), and H2d (β = 0.471, t-value = 5.753) are supported. Ethical responsibility in CSR is not found to have an influence on corporate image (β = 0.085, t-value = 1.249); thus, H2d is not supported. Furthermore, customer trust was also found to have a significant impact on corporate image (β = 0.178, t-value = 3.504).

This study applied activity theory by Kofod-Petersen & Cassens [86] and the indirect effects as indicated in Table 5, generated by Smart pls to test the mediation results. According to the results shown in Table 5, customer trust fully mediates the relationship between economic and legal CSR with corporate image, while it partially mediates ethical and philanthropic CSRs’ relationship with corporate image. 

## 5. Discussion

This study is linked to the success of the hospitality marketing strategy; hence, it was imperative to perform an in-depth examination of the corporate image of the hotel. This study provides the framework to describe how the hotel’s corporate image and customers’ trust can be impacted by the four dimensions of corporate social responsibility. This framework will support other scholars in the hotel industry to comprehend customers’ conduct built on their experiences of multidimensional CSR initiatives, corporate image, and customer trust.

With the growth of corporate social influence, CSR initiatives have been considered as a crucial component for a company’s management. There is a requirement to recognize customers’ opinions of CSR behaviors for potential corporate profitability. Consequently, this research examines the impact of multidimensional corporate social responsibility initiatives on corporate image. The findings revealed persuasive evidence stating customers’ viewpoints regarding the CSR initiatives of a company could encourage their trust in the company. Each of the CSR constructs was found to have a significant impact on corporate image except, philanthropic CSR. The results of this study are somewhat similar to the study by Kim et al. [87]. According to their findings, ethical, economic, philanthropic, and legal responsibility, influenced corporate image and customer’s citizenship behavior. The outcomes are also consistent with the generally recognized belief [15,88] that CSR significantly impacts the corporate image. However, in terms of the insignificant relationship of philanthropic CSR and CI, this study’s results are somewhat similar to the research study by Park et al. [15]. According to their study, a company’s ethical responsibilities did not have a significant impact on the CI of the company. This study delivers empirical inferences for businesses by validating the result of CSR initiatives as a principal factor in developing customers’ trust as an organizational objective in the hotel business. It implies that the higher a company is understood to be involved in behaviors such as establishing a reasonable pricing strategy and generating jobs, the greater its corporate image is perceived by the customers. Additionally, companies’ voluntary endeavors in favor of ecological sustainability and civil rights generate a positive attitude and a decent image. Furthermore, customers tend to have a constructive image of a business that conforms with rules and laws. Finally, a company requires to meet the expectation of the customers, perceiving the company to be a decent representative of society by partaking in or supporting social wellbeing. The outcomes indicate that to reinforce the corporate image, companies must give more consideration to the economic, legal, and ethical dimensions of corporate social responsibility.

According to the results, all CSR constructs had a significant impact on customer trust, except ethical responsibility. The research outcomes are consistent with the previous studies in the framework of customers’ environmental activities [48] concerning the constructive influence of customers’ corporate social responsibility experiences on their trust. When customers consider that a hotel worries about the outcomes of its functions on the environment and the community, they deem the hotel as a benevolent business, and they will trust it more. A study by Shafieizadeh & Tao [66] examined the impact of customers’ views of a restaurant menu that comprises local food knowledge and its fundamental mechanism. The outcomes revealed that assessments of menu information influenced customers’ views regarding the restaurant’s openness and CSR initiatives significantly and additionally affected customers’ trust significantly, but indirectly, via perceived corporate social responsibility actions. However, the insignificant relationship of ethical responsibility and trust was not found to be similar with other research studies. Previous researches found ethical responsibility to be in a significant relationship with customer trust [54,89]. 

Furthermore, the results of this study showed a positive and significant relationship between corporate image and customer trust. A study by Triatmanto et al. [60] revealed how the experiences and beliefs of hotel management in East Java enhance the corporate image as a marketing strategy of a hotel. Managers of the hospitality industry were selected as research respondents for this study. The findings demonstrated that the hotel management should comprehend the hotel’s corporate image to maintain customers’ trust in the hotel. Corresponding to the view of hotel management corporate image is exhibited by the trust presented by the hotel [90,91]. The managers in the hospitality industry also think that the hotel can acquire many guests if the customers trust the hotel. This implies that each hotel should be able to develop psychological recognition in the opinions of customers so that they can be driven to experience the same hotel again [64]. 

This research intended to build an enhanced understanding of the connection between corporate social responsibility and corporate image by contemplating a broader variety of CSR programs and the position of customer trust as a mediator between the relationship of corporate social responsibility and corporate image. Results showed that amongst the four types of CSR programs, the company’s activities of economic and legal responsibility were fully mediated by customer trust, while ethical and philanthropic responsibility were partially mediated by customer trust. These outcomes are somewhat similar to the studies of Jongchul et al.’s [92] and Yoon et al.’s [88], according to results customers have a tendency to attach intentions to the company’s philanthropic initiatives, and that a view of honesty and trustworthiness in the motivations results in a constructive company assessment.

## 6. Theoretical Implications

The research has the following theoretical implications. The first is that despite having theoretical assistance for the association of corporate social responsibility and corporate image in numerous service fields, little investigations have empirically examined the impacts of CSR’s four dimensions on corporate image and customers’ trust in association with the hotel service industry. This study is unique from earlier CSR research based on an assessment of the connection between CSR dimensions and corporate image to examine customers’ trust in the company. Moreover, the dimensional composition of hotel corporate social responsibility was verified by examining the range of validity and reliability. Furthermore, the corporate social responsibility scale was demonstrated to be efficient in calculating the customers’ attitude, trust, and perception regarding the service industry. Therefore, this scale might be utilized to describe additional variables, including customers’ pro-social conduct, loyalty, and post-purchase conduct. Hence, the findings of this research can assist to build innovative information and expand to subsequent studies [1]. 

This research indicates that a company’s corporate social responsibility initiatives affect the corporate image, which additionally affects the development of trust among customers and companies. Via the evaluation of the framework of CSR, customers’ trust and corporate image were observed to be vital to a workable relationship. This study can hence be a beginning point in examining the association between corporate social responsibility and corporate image in customers’ trust. It is assumed that customers recognize a company because its features are expressive, or parallel to their individual features or the features that they seek to possess. It is found that corporate social responsibility constantly plans a business’s philanthropical and considerate principles that underline the uniqueness or character of the company, therefore it could be utilized as a powerful instrument by companies to create convincing images that appeal to customers’ societal and emotional wants and encourage trust to a service company [3,93]. Certainly, the present research findings discovered that corporate social responsibility strengthens the customers’ identification with a business in addition to their trust in that business. This conclusion develops the opinion that the blend of both viewpoints proposes an additional combined and complete method to understand the path from corporate social responsibility to the loyalty of customers, therefore offering managers with an added insight regarding the role and value of corporate social responsibility in developing trustworthy associations with customers.

Consequently, it was feasible to evaluate factors that impact corporate social responsibility and corporate image. By the formation and confirmation of subfactors of corporate social responsibility and corporate image, it facilitates to comprehend the four types of CSR and corporate image dimensions that impact customers’ trust. A company’s economic, legal and ethical CSR have a major effect on the corporate image of that company. Corporate image has an impact on making a suggestion, and that suggestion has an impact on the creation of customers’ trust. This research improves the current information by demonstrating the mediation impact of customers’ trust on the association between economic and legal CSR, and corporate image. Once customers consider that a hotel is striving to be understandable to them, by offering comprehensive information regarding the hotel’s actions, they believe that it appreciates the expectations of its stakeholders, and hence the customers trust the hotel more.

## 7. Managerial Implications

The industrial and managerial implications of this research study are as follows. Hotels must try to enhance their corporate image, as the image is impacted by both the four dimensions of CSR and customers’ trust. Corporate social responsibility was found to have an effect on corporate image, and economic, legal, and ethical corporate social responsibility initiatives were found to have a significant impact on corporate image whereas philanthropic responsibilities had no significant impact on corporate image. Based on this conclusion, operators can concentrate on economic CSR to boost the corporate image and promote customers’ trust. Economic CSR initiatives produce profit via the delivery of attractive services. Customers wish for quality services at a satisfactory price. Firms can meet economic CSR initiatives by offering services much better than customers’ beliefs. A company’s economic initiatives mostly ignore the ecosystem or social responsibilities. Economic corporate social responsibility initiatives must hence be endorsed for profit-making purposes as a marketing strategy and an attractive answer to the social and ecological difficulties faced by a company. The hotel industry is engaged in organizing environmentally friendly marketing related to the ethical responsibilities to avoid pollution in the environment. Regarding legal responsibilities, the hotel industry must conform with sanitation and security procedures. Ethical and legal corporate social responsibility initiatives are considered to be vital, particularly in a competitive market. To satisfy philanthropic CSR, firms have maintained to organize charitable events or sponsor donations besides giving extra benefits to their employees. Businesses are now needed to engage with their society and give earnings back to society. 

The results of this study can be utilized by employees to promote customers’ trust, which can be useful to businesses. Maintaining constructive connections with customers can assist a business. Businesses want optimistic communications with customers to boost customers’ trust. A business can build a community to accelerate the interaction between customers. Furthermore, if customers are paid with a souvenir or a coupon for their work and time, they may gain an emotional inclination to assist the business. As a consequence, customers will be extra loyal to the business. 

Managers ought to be careful and tactical in using ethical and philanthropic responsibilities. Once a company performs philanthropic initiatives for a worthy reason, the company expects that customers consider a constructive acknowledgment regarding the company’s motivations. Though businesses must contemplate the optimistic impacts of corporate social responsibility on their employees with discretion, as they might also possess some detrimental adverse outcomes like the staffs’ inclination to engage in extra hard work and therefore result in labor dependence [94]. Accountable entrepreneurship is an enduring action and its initiation into the business delivers even greater advantages when there is an awareness for these initiatives [95,96].

This study made further practical contributions in offering hotels management understandings into the insight of corporate social responsibility dimensions and evaluating a hotel’s corporate social responsibility operations. Consequently, hotel commerce must signify the necessity of smart customers and society, alongside worldwide developments regarding environmental safety. A hotel can comprehend its competition by frequently assessing and evaluating its corporate social responsibility endeavors with other hotels. Moreover, corporate social responsibility initiatives would additionally help enable internal organizational marketing by keeping decent relationships with personnel [1]. Service managers need to concentrate on corporate image protection and take into attention the significance of a corporation’s image for their shareholders. More precisely, by skillfully controlling corporate image and corporate social responsibility as the prediction/interaction instrument of the company, they can encourage business relationships that may stimulate long-term relations and trust with customers [3,97].

## 8. Limitations and Future Research

This research has some shortcomings that have the possibility for potential research. The survey of this research was conducted in Pakistan; hence it has a shortage of representativeness and self-selection bias. To produce more generalized conclusions, the research survey can be done in numerous countries. Moreover, Pakistan is an emerging economy, future research can be conducted in developed economies and their results can be compared. Corporate image is an individual construct, therefore for a thorough investigation in the service perspective, several attitudinal and cognitive antecedents of corporate social responsibility should be considered for future potential research. 

Furthermore, although Carroll’s four CSR dimensions have been used recently in the service industry research [98,99]. Carroll’s four corporate social responsibility dimension model has been extensively presented in the studies related to the hotel industry. Nevertheless, the hotel business varies from other businesses in conditions of stakeholders, customers, policies, organizational culture, and products due to its distinctive qualities that are indivisible, and intangible [100]. Additionally, the business neglects to indicate an expanding understanding of ecology, sustainability, renewable resource, and ecological safety [1,101,102,103,104]. Hence, future researchers can utilize the modified version of Carroll’s CSR model while studying the service industry 

Finally, since customers can act as effective agenda planners via various online platforms, they can become key influencers in times of crisis. Hence, future researchers can target the impact of customers communication on the periods of pre and post-crisis situations related to CSR.

## 9. Conclusions

The purpose of this is to analyze the connection between CSR dimensions and corporate image to examine customers’ trust in an emerging economy, especially in times of crisis. This research empirically examines the relationships between a multi-dimensional set of corporate social responsibility (CSR) initiatives, numerous dimensions of customer trust, and corporate image in an emerging economy. It also analyzes the mediating effect of customer trust on the relationship between CSR and corporate image. The results revealed that economic, legal, and ethical CSR significantly impacted corporate image, while philanthropic CSR did not affect the corporate image. However, economic, legal, and philanthropic CSRs were found to be in a significant relationship with customer trust, while ethical CSR was not in a significant relationship with customer trust. Finally, customer trust fully mediated the relationship between economic and legal CSR with corporate image, whereas it partially mediated the relationship between ethical and philanthropic CSR. 

## Figures and Tables

**Table 1:** Convergent validity.

Construct	Item Code	Factor Loading	Cronbach’s Alpha	Composite Reliability	Average Variance Extracted (AVE)
Economic CSR	ECO1 ECO2 ECO3 ECO4	0.821 0.883 0.876 0.862	0.884	0.920	0.741
Legal CSR	LEG1 LEG2 LEG3	0.833 0.889 0.879	0.835	0.901	0.753
Ethical CSR	ETHI1 ETHI2 ETHI3	0.888 0.924 0.891	0.885	0.929	0.813
Philanthropical CSR	PHI1 PHI2 PHI3	0.803 0.790 0.675	0.870	0.920	0.793
Corporate image	CI1 CI2 CI3 CI4 CI5 CI6 CI7 CI8 CI9 CI10	0.735 0.756 0.809 0.786 0.830 0.832 0.745 0.705 0.719 0.759	0.923	0.935	0.591
Integrity	INT1 INT2 INT3 INT4	0.870 0.917 0.924 0.918	0.929	0.949	0.824
Expertise	EXP1 EXP2 EXP3	0.898 0.936 0.907	0.901	0.938	0.835
Benevolence	SOC1 SOC2 SOC3	0.911 0.909 0.851	0.870	0.920	0.794
Customer trust	Integrity Expertise Benevolence	0.926 0.907 0.866	0.944	0.952	0.666

**Table 2:** Standardized factor loadings and cross-loadings of the outer model.

	Economic (CSR)	Ethical (CSR)	Legal (CSR)	Philanthropic (CSR)	Customer Trust	Corporate Image
ECO1	0.821	0.249	0.344	0.298	0.293	0.432
ECO2	0.883	0.247	0.403	0.320	0.326	0.502
ECO3	0.876	0.291	0.378	0.354	0.382	0.565
ECO4	0.862	0.321	0.433	0.374	0.405	0.595
ETHI1	0.298	0.888	0.477	0.622	0.540	0.508
ETHI2	0.263	0.924	0.507	0.643	0.546	0.535
ETHI3	0.317	0.891	0.596	0.696	0.567	0.594
LEG1	0.468	0.562	0.833	0.677	0.642	0.740
LEG2	0.391	0.505	0.889	0.665	0.599	0.669
LEG3	0.318	0.452	0.879	0.681	0.638	0.635
PHI1	0.342	0.696	0.714	0.905	0.652	0.629
PHI2	0.369	0.630	0.670	0.893	0.688	0.650
PHI3	0.342	0.619	0.696	0.874	0.726	0.663
EXP1	0.328	0.495	0.612	0.657	0.816	0.592
EXP2	0.311	0.499	0.598	0.633	0.838	0.579
EXP3	0.341	0.458	0.570	0.605	0.833	0.545
INT1	0.313	0.533	0.587	0.634	0.812	0.608
INT2	0.332	0.433	0.578	0.590	0.829	0.587
INT3	0.347	0.456	0.575	0.606	0.860	0.560
INT4	0.348	0.561	0.634	0.663	0.862	0.625
SOC1	0.375	0.510	0.538	0.624	0.735	0.574
SOC2	0.368	0.494	0.598	0.641	0.768	0.590
SOC3	0.316	0.552	0.608	0.666	0.804	0.604
CI1	0.490	0.521	0.650	0.567	0.537	0.735
CI2	0.590	0.462	0.663	0.598	0.590	0.809
CI3	0.584	0.456	0.568	0.542	0.563	0.786
CI4	0.567	0.462	0.638	0.572	0.581	0.830
CI5	0.519	0.446	0.597	0.568	0.559	0.832
CI6	0.456	0.522	0.613	0.610	0.554	0.745
CI7	0.363	0.418	0.495	0.453	0.493	0.705
CI8	0.325	0.406	0.533	0.476	0.458	0.719
CI9	0.343	0.435	0.646	0.551	0.518	0.759
CI10	0.441	0.519	0.633	0.624	0.645	0.756

Note 1: ECO CSR, Economic CSR; ETHI CSR, Ethical CSR; LEG CSR, Legal CSR; PHI CSR, Philanthropic CSR; EXP, Expertise trust; INT, Integrity trust; SOC, Social Benevolence; CI, Corporate Image.

**Table 3:** Hypotheses results for model A.

Hypothesis	Path Coefficient	T Values	*p* Values	Results
H1a: Economic (CSR) -> Corporate image	0.010	0.297	0.766	Not Supported
H1b: Legal (CSR) -> Corporate image	0.589	7.805	0.000	Supported
H1c: Ethical (CSR) -> Corporate image	0.120	2.191	0.029	Supported
H1d: Philanthropic (CSR) -> Corporate image	0.159	2.304	0.042	Supported

**Table 4:** Hypotheses results for Model B.

Hypothesis	Path Coefficient	T Values	*p* Values	Results
H1a: Economic (CSR) -> Corporate image	0.293	6.080	0.000	Supported
H1b: Legal (CSR) -> Corporate image	0.394	6.198	0.000	Supported
H1c: Ethical (CSR) -> Corporate image	0.106	2.332	0.020	Supported
H1d: Philanthropic (CSR) -> Corporate image	0.091	1.323	0.186	Not Supported
H2a: Economic (CSR) -> Customer trust	0.076	1.936	0.053	Supported
H2b: Legal (CSR) -> Customer trust	0.272	3.627	0.000	Supported
H2c: Ethical (CSR) -> Customer trust	0.085	1.249	0.212	Not Supported
H2d: Philanthropic (CSR) -> Customer trust	0.471	5.761	0.000	Supported
H3: Customer trust -> Corporate image	0.178	3.504	0.000	Supported

**Table 5:** Hypotheses and Mediating effects.

Hypothesis	Indirect Effects	Mediation
Economic (CSR) -> customer trust -> corporate image	0.014	Full
Legal (CSR) -> customer trust -> corporate image	0.048	Full
Ethical (CSR) -> customer trust -> corporate image	0.015	Partial
Philanthropic (CSR) -> customer trust -> corporate image	0.084	Partial

## Appendix A

**Scales**

**Corporate Social Responsibility**

- The hotel improves the tourism industry.
- The hotel generates employment through its operations.
- The hotel strives to activate the local economy.
- The hotel strives to achieve sustainable growth.
- The hotel properly implements health and safety rules and regulations.
- The hotel has established appropriate regulations for customers to abide by.
- The hotel strives to abide by regulations related to its customers’ well-being.
- The hotel has established ethical guidelines for business activities.
- The hotel tries to become an ethically trustworthy company.
- The hotel makes efforts to fairly treat customers.
- The hotel participates in a variety of volunteer activities by starting the company’s volunteer group.
- The hotel supports social welfare projects for the underprivileged.
- The hotel supports education programs.

**Corporate Image (CI)**

- I think the hotel emphasizes the rights of customers.
- I have good impressions of the hotel.
- In my opinion, the hotel has a good image in the minds of consumers.
- I think the service value provided by the hotel is high.
- I think the employees are very friendly.
- The appearance of the hotel is appealing.
- The area around the hotel is clean.
- The hotel is located in a nice area.
- The prices at the hotel are fair.
- I obtain value for my money at the hotel.

**Consumer Trust**

- The hotel performs its role of providing hoteling services very well.
- Overall, the hotel is a capable and proficient hoteling services provider.
- In general, the hotel is deeply knowledgeable about customer care.
- The hotel is truthful in its dealings with me.
- I would characterize the hotel as honest.
- The hotel would keep its commitments.
- The hotel is sincere and genuine.
- I believe that the hotel would act in my best interest.
- If I required help, the hotel would do its best to help me.
- The hotel is interested in my well-being, not just its own.
